# Proline-rich acidic protein 1 upregulates mitotic arrest deficient 1 to promote cisplatin-resistance of colorectal carcinoma by restraining mitotic checkpoint complex assembly

**DOI:** 10.7150/jca.84048

**Published:** 2023-05-21

**Authors:** Jintian Song, Yigui Chen, Hui Yu, Liang Zheng, Yi Wang, Dan Li

**Affiliations:** 1 Department of Gastroenterology, Fujian Medical University Union Hospital, Fuzhou 350001, Fujian Province, China.; 2 Department of Abdominal Oncology, Clinical Oncology School of Fujian Medical University, Fujian Cancer Hospital, Fuzhou, 350014, Fujian Province, China.; 3 Fujian Medical University, FuzhouCity 350000, Fujian Province, Fujian Province, China.; 4 Department of pharmacy, Clinical Oncology School of Fujian Medical University, Fujian Cancer Hospital, No. 420, Fuma Rd., Jin'an District, Fuzhou City 350014, China.; 5 Fujian Clinical Research Center for Digestive System Tumors and Upper Gastrointestinal Diseases, Fujian Medical University, Fuzhou 350001, Fujian Province, China.

**Keywords:** Proline-rich acidic protein 1, mitotic arrest deficient 1, spindle assembly checkpoint, colorectal carcinoma, cisplatin resistance

## Abstract

**Background:** The mechanism underlying cisplatin resistance in colorectal carcinoma (CRC) has not yet been elucidated. This study is aimed to illustrate the indispensable role of proline-rich acidic protein 1 (PRAP1) in cisplatin-resistant CRC.

**Methods:** Cell viability and apoptosis were monitored using cell counting kit-8 and flow cytometry. Immunofluorescence and morphological analysis were used to determine mitotic arrest in cells. *In vivo* drug resistance was evaluated using a tumor xenograft assay.

**Results:** PRAP1 was highly expressed in cisplatin-resistant CRC. PRAP1-upregulation in HCT-116 cells increased chemoresistance to cisplatin, whereas RNAi-mediated knockdown of PRAP1 sensitized cisplatin-resistant HCT-116 cells (HCT-116/DDP) to cisplatin. PRAP1-upregulation in HCT-116 cells hindered mitotic arrest and the formation of mitotic checkpoint complexes (MCC), followed by an increase in multidrug-resistant proteins such as p-glycoprotein 1 and multidrug resistance-associated protein 1, while PRAP1-knockdown in HCT-116/DDP cells partly restored colcemid-induced mitotic arrest and MCC assembly, resulting in decreased multidrug-resistant protein levels. PRAP1 downregulation-mediated sensitization to cisplatin in HCT-116/DDP cells was abolished by the inhibition of mitotic kinase activity by limiting MCC assembly. Additionally, PRAP1-upregulation increased cisplatin-resistance in CRC *in vivo*. Mechanistically, PRAP1 increased the expression of mitotic arrest deficient 1 (MAD1), that competitively binds to mitotic arrest deficient 2 (MAD2) in cisplatin-resistant CRC cells, leading to failed assembly of MCC and subsequent chemotherapy resistance.

**Conclusion:** PRAP1-overexpression caused cisplatin resistance in CRC. Possibly, PRAP1 induced an increase in MAD1, which competitively interacted with MAD2 and subsequently restrained the formation of MCC, resulting in CRC cells escape from the supervision of MCC and chemotherapy resistance.

## Introduction

Colorectal carcinoma (CRC), one of the most common gastrointestinal cancers, poses a threat to people 1. In recent decades, the incidence of CRC has continued to increase rapidly worldwide, and the mortality rate is also increasing annually at the rate of 2% 2, with an approximately 10% survival rate 3. CRC still represents a strenuous burden worldwide, despite advances in its therapy 4, 5. Chemotherapy and targeted therapy are usually combined with antitumor drugs, including cisplatin, 5-Fluorouracil (5-FU), and capecitabine in the clinical treatment of CRC 6. Unfortunately, resistance of patients with CRC to chemotherapy drugs is one of the biggest obstacles to end-stage chemotherapy 7. However, the exact mechanism underlying chemotherapy resistance in CRC remains unclear.

Chromosomal instability plays an important role in the development of multiple cancers 8. The accurate separation of chromosomes and genetic materials is essential for cell division 9. Chromosomal instability caused by chromosome addition or deletion eventually affects the orderly cell cycle 10-12. Mitotic checkpoint, also called the spindle assembly checkpoint (SAC), is one of the most prevalent causes of chromosomal aberrations 13, 14. SAC ensures the correct attachment of chromosomes to spindle fibers using mitotic checkpoint complex (MCC) 15, 16. MCC is composed of many genes, including cell division cycle protein 20 (Cdc20), mitotic arrest deficient 2 (MAD2), budding uninhibited by benzimidazole-related 1 (BUBR1), and budding uninhibited by benzimidazoles 3 (Bub3), which regulate the SAC process 17, 18. SAC and MCC are the key components for maintaining the stability of the cell genome, and functional defects of SAC lead to chromosome polyploidy, which is the initial step for tumor cells to develop drug resistance 19. With advances in research, it is widely accepted that SAC inhibition caused by MCC abnormalities in cancer cells represents the potential resistance mechanism 20. Therefore, abnormal mitotic checkpoint signals are among the main causes of CRC and chemotherapy resistance.

Proline-rich acidic protein 1 (PRAP1), is a p53-responsive gene induced by genotoxic stress 21. PRAP1, which contains functional p53-response elements, can regulate tumor cell growth 22. Knockdown of PRAP1 advances the sensitivity of cells to 5-FU and protects cells from apoptosis by inducing cell cycle arrest 23. Researchers have designed a combination drug of cisplatin and chlorambucil that can partly overcome cisplatin resistance to enhance DNA damage and antitumor activity via inhibition of PRAP1 24. Accordingly, PRAP1 is involved in the regulation of chemotherapy resistance in tumor cells. Remarkably, PRAP1 can reduce the expression of mitotic arrest deficient 1 (MAD1), a key constituent of SAC signaling, and suppress mitotic checkpoint signaling in hepatocellular carcinoma 25. It is possible that PRAP1 may regulate the activities of MCC members to enable SAC signaling and may participate in the process of chemotherapy resistance.

Therefore, we hypothesized that PRAP1 integrates with MAD1 to regulate MCC activity. In this study, we aimed to illustrate the indispensable role of PRAP1 in cisplatin-resistant CRC. We demonstrated the expression pattern and biological function of PRAP1 in patients with CRC and cisplatin-resistant CRC cells. Mechanistically, the roles of MCC-mediated mitotic arrest and SAC activity were further investigated in cisplatin-resistant CRC cells. Collectively, our findings provide novel therapeutic strategies for improving the clinical status of patients with CRC exhibiting cisplatin resistance.

## Materials and methods

### Data collection and analysis

The transcriptional expression of PRAP and MAD1 in human colon adenocarcinoma (COAD) specimens and normal tissues was analyzed using the Gene Expression Profiling Interactive Analysis (GEPIA) version 2 database 26. The expression profiles of *PRAP1* and *MAD1* in drug-treated CRC cell lines and PRAP1 expression status in non-recurrence and recurrence patients were obtained from the Oncomine database. The expression association between *PRAP1* and *MAD1* was analyzed using the Encyclopedia of RNA Interactomes (ENCORI) Starbase 27.

### Cell culture

Human CRC cell lines, including HCT-116, HT-29, Lovo, and SW480; cisplatin-resistant human CRC cell lines HCT-116/DDP and HT-29/DDP; 5-FU-resistant human CRC cell line HCT-116/5-FU; and normal colorectal cell line CRL-1790 were purchased from the American Type Culture Collection (ATCC, Manassas, USA). All cells were maintained in RPMI-1640 medium (R8758, Sigma, USA) supplemented with 10% fetal bovine serum (FBS, C0232, Gibco, USA) containing 1% penicillin/streptomycin (10378016, Thermo, USA). All cell lines were cultured at 37℃ in a humidified atmosphere containing 5% CO_2_.

### Cell transfection and treatment

The CDS fragments of *PRAP1* and *MAD1* were subcloned into the pEGFP-N1 vector plasmid, confirming the correctness of the recombinant plasmid sequences. Plasmids were transfected into cells using Lipofectamine 3000 (L3000001, Thermo, USA) for 24 h according to the manufacturer's instructions. Sequence synthesis of siPRAP1 (5'-CCGGTTGTGGGTGATGCCAAA-3') and the negative control (siNC, 5'-CCGATTATGGGTAATGCCGAA-3') were performed by GenePharma (Shanghai, China) and were transiently transfected into cells for 24 h. Different concentrations of cisplatin (5 μM,10 μM and 20 μM; A10221, Adooq Bioscience, USA) were chosen to induce cells for 24, 48, 72, and 96 h. For the mitotic arrest assay, treated or control cells were incubated with 0.1 μg/mL colcemid (C3915, Sigma, USA) or the combination of colcemid and 10μM/mL Mps1-IN-1 (HY-13298, MCE, USA) for 24 h. Mps1-IN-1 was used to inhibit the formation of MCC complex.

### Western blotting analysis

Total cell protein was extracted using lysis buffer containing a protease inhibitor cocktail (P8849, Merck, USA) and phosphatase inhibitors (P2850, Merck, USA). The protein concentration was measured using a BCA Protein Quantitative Kit (BCA1, Merck, USA). Equal amounts of protein were loaded onto a 10% SDS-PAGE gel and then blotted onto polyvinylidene fluoride membranes (IPFL00010, Merck, USA). After blocking in the 5% non-fat milk for 1 h, the bands were incubated with primary antibodies at 4℃ overnight. The primary antibodies used were as follows: PRAP1 (H00117177, Abnova, USA, 1:1000), Cdc20 (ab183479, Abcam, USA, 1:1000), MAD1 (ab184560, Abcam, USA, 1:500), MAD2 (ab10691, Abcam, USA, 1:1000), Bub3 (sc-376506, Santa Cruz, USA, 1:500), BUBR1 (sc-47744, Santa Cruz, USA, 1:500), p-glycoprotein 1 (MDR1, sc-55510, Santa Cruz, USA, 1:500), multidrug resistance-associated protein 1 (MRP1, ab24102, Abcam, USA, 1:1000), and reduced glyceraldehyde-phosphate dehydrogenase (GAPDH, ab9485, Abcam, USA, 1:5000). On the next day, membranes were incubated with homologous secondary Goat Anti-Mouse IgG (H+L) horseradish peroxidase (HRP, S0002, Affinity, USA, 1:5000) or Goat Anti-Rabbit IgG (H+L) HRP (S0002, Affinity, USA, 1:5000) for 1 h at room temperature. Membranes were visualized using a chemiluminescence western blotting system and quantified using Image J (1.8.0, National Institutes of Health, USA) software.

### Quantitative real-time polymerase chain reaction (RT-PCR)

Total RNA was isolated from cultured cells and first-strand cDNA was synthesized using One-Step gDNA Removal and cDNA Synthesis SuperMix (AH311-02, TransScript, CN). PCR analysis of cDNA was conducted using SYBR Green One-Step qRT-PCR SuperMix (AQ211-01, TransScript, CN) according to the manufacturer's recommendations. The primer sequences are presented in **Table 1**. GAPDH served as the internal control. mRNA levels were analyzed by linear amplification in a PCR instrument (Bio-Rad, USA) and calculated using the 2^-ΔΔCt^ method.

### Mitosis Analysis

Control cells or different plasmid-transfected cells (n=30,000) were implanted onto 6-well plates for 24 h. Subsequently, the cells were treated with 0.1 μg/mL colcemid (C3915, Sigma, USA) for 16 h. Images were obtained using Ptychographic Quantitative Phase Imaging technology by Livecyte Cell Analysis System (Phasefocus, UK) at the indicated time points. The rounded-up and bright cells were considered to be in the mitotic arrest phase.

### Immunofluorescence

Cells were grown on chamber slides overnight with additional treatment. The cells were then fixed in 4% paraformaldehyde (PFA, 30525-89-4, Merck, USA) for 10 min, followed by permeabilization with 0.1% Trionx 100 (85111, Thermo Fisher, USA) for 5 min. After blocking in 5% BSA for 15 min, the slides were incubated with phosphorylated histone 3 (pH3) antibody (ab8580, Abcam, USA, 1:50) at 4℃ overnight. The next day, the slides were incubated with a fluorescent IgG secondary antibody for 1 h in the dark. The cells were then stained with Hoechst 33258 staining dye solution (ab228550; Abcam, USA) to visualize the nuclei. Mitotic-arrested cells were pH3-positive. The fluorescence signals were photographed using a Nikon T1E confocal microscope.

### Cell Counting Kit-8 (CCK8) Assay

Cell viability was evaluated using the CCK8 Assay kit (HYK0301, MCE, USA). Briefly, control HCT-116 and HCT-116/DDP cells or siRNA/plasmid-transfected cells were seeded at a density of 2,000 cells/well in 96-well plates. Cells were then treated with different concentrations of cisplatin (5, 10, and 20 μM) for 24, 48, 72, and 96 h. Next, 10 μL CCK8 solution was added to each well for additional 4 h incubation at 37°C. Subsequently, the absorbance values at 450 nm were determined using a spectrophotometer.

### Apoptosis Assay

An Annexin V-FITC cell apoptosis detection kit (APOAF-20TST, Merck, USA) was used to assess cell apoptosis. In brief, cells were washed with phosphate buffer solution (PBS), resuspended in AnnexinV-FITC solution for 10 min, and incubated with binding buffer for 20 min at 37℃ in the dark. Finally, the percentage of apoptotic cells was analyzed using a flow cytometer.

### Co-Immunoprecipitation (Co-IP)

Total protein was obtained from the cell precipitate using RIPA lysis buffer (R0010, Solarbio, CN) containing 1% protease inhibitor cocktail and 1 mM phenylmethanesulfonyl fluoride (PMSF) protease inhibitor (36978, Thermo, USA). Subsequently, extracted protein and 50 mL Protein A Agarose Beads (9863, CST, USA) were fully reacted for 4 h at room temperature, and then the admixture was incubated with primary antibodies at 4℃ overnight. The following day, the saturated immunoprecipitants were washed and collected for western blotting.

### Tumor xenograft

Male BALB/c nude mice (5-6 weeks of age, 16-18 g) were purchased from the Laboratory Animal Center of Xiamen University, China. Mice were maintained in a specific-pathogen-free (SPF) room under controlled temperature (20±2°C) and humidity, with free access to food and water. All experimental procedures were approved by the Animal Welfare Committee of the Research Organization of Fujian Cancer Hospital (2017-073-01). Subsequently, a xenograft model was established by subcutaneous injection of 0.2 mL (2 X 10^6^) HCT-116-EGFP control cells and PRAP1-stable expression cells. The cells were resuspended in serum-free RPMI-1640. Once the tumor volume grew to approximately 0.75 cm^3^ (day 15 after cell injection), mice bearing tumor were randomly divided into four groups (n=6 per group). Two groups of control HCT-116 cells-bearing mice or two groups of PRAP1-stable expression cells-bearing mice were treated orally with normal saline and cisplatin (6 mg/kg/day), and housed for an additional 8 consecutive days. The tumor size was measured on days 9, 11, 13, 15, 17, 19, 21, and 23 after the initial injection. Tumor volume was monitored using the formula: Volume=0.5xaxb^2^, where a and b indicate the length and width of the tumor, respectively. After modeling, all mice were sacrificed by cervical dislocation and the tumors were harvested. After weighing, all tumors were stored at -80 °C until analysis.

### Immunohistochemical staining

Eleven fresh CRC tissues (n=11) and the corresponding para-carcinoma tissue (n=11) were collected from Fujian Cancer Hospital. The enrolled patients who was not suitable for radiotherapy, radiofrequency and other local treatment were confirmed as colorectal adenocarcinoma with histologically or cytologically. Informed consent was obtained from all subjects, and the study was approved by the ethics committee of Fujian Cancer Hospital. Patients who received systemic therapy (such as chemotherapy, immunotherapy, targeted therapy, etc.) or local chemotherapy (including endovascular chemotherapy, arterial infusion chemotherapy, etc.) and patients with systemic disease were excluded. Tumor tissues were embedded in paraffin and 5 μm tissue sections were cut. The tissue slices were dewaxed in xylene and hydrated in graded ethanol solutions. Antigen retrieval was performed in an autoclave for 5 min, followed by incubation with 0.3% H_2_O_2_ for 20 min. After blocking with 1% bovine serum albumin for 1 h, the slices were incubated overnight with primary antibodies against Ki67 (ab15580, Abcam), PRAP1 (HPA038713, Sigma, UK, 1:500), and MAD1 (ab184560, Abcam, USA, 1:100). The next day, tissue sections were hybridized with the secondary antibody and subsequently subjected to a chromogenic reaction using 3,3'-Diaminobenzidine Tetrahydrochloride (DAB) reagent (Zhongshan Biotech, Peking, CN). Five high-power fields of each sample were chosen by three independent pathologists to evaluate the relative Ki67-positive cell number using Image Pro Plus software v6.0 (Media Cybernetics, Inc. Maryland, USA).

### Statistical Analysis

Data are reported as the mean ± S.D. All statistical data were analyzed using GraphPad software (version 9.0; La Jolla, CA, USA). Comparisons between different groups were performed using an unpaired Student's two-tailed t-test or one-way ANOVA. P<0.05.

## Results

### High expression of PRAP1 is associated with chemotherapy resistance of CRC

**Figure 1:**
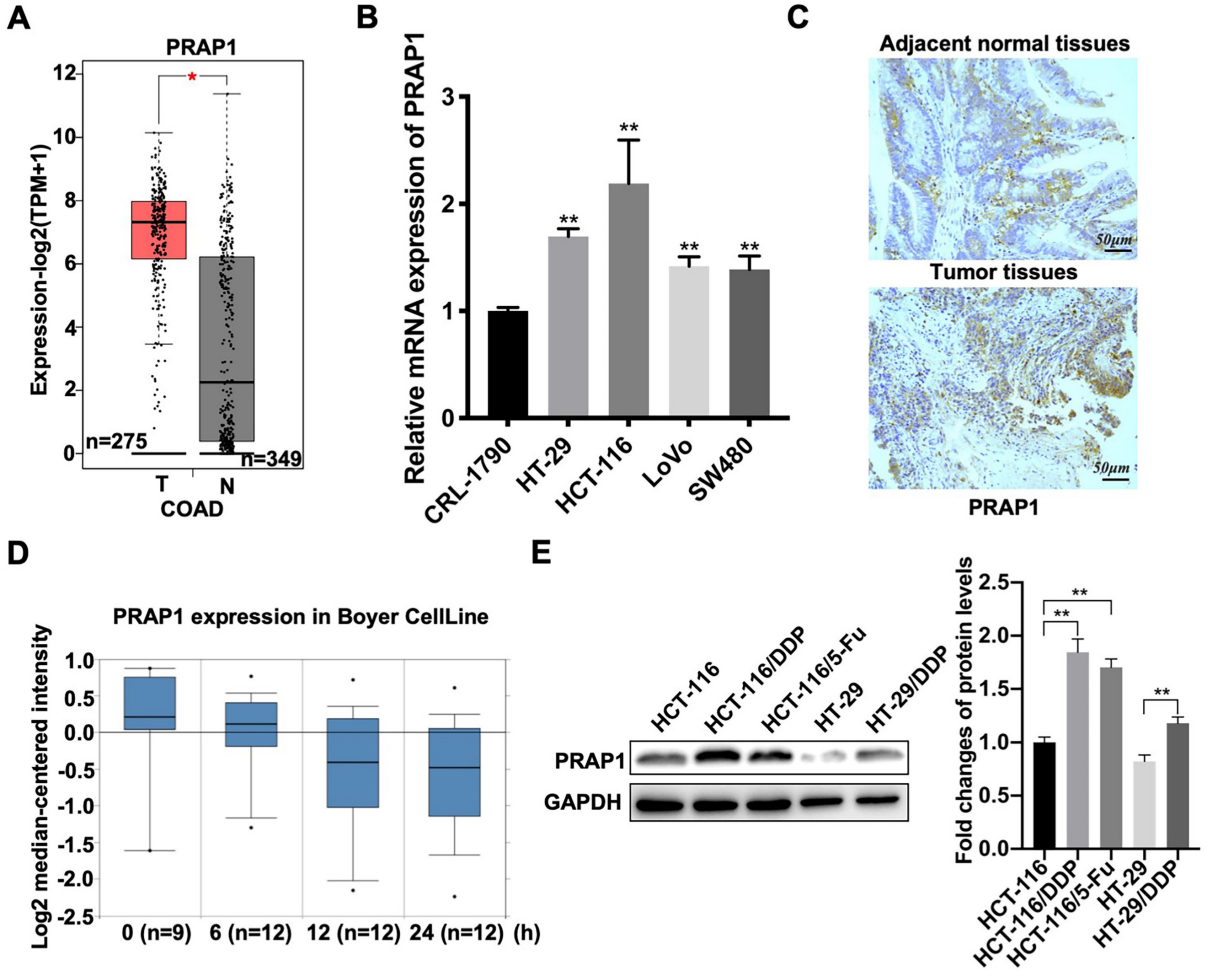
** Expression pattern of proline-rich acidic protein 1 (PRAP1) in human colorectal cancer. (A)** PRAP1 expression in clinical colorectal carcinoma (CRC) tissues (n=275) and control group (n=349) was detected by using Gene Expression Profiling Interactive Analysis (GEPIA 2). **(B)** The mRNA expression of PARP1 in several CRC cell lines including HCT-116, HT-29, Lovo and SW480 and normal colon cell line CRL-1790 were determined by qRT-PCR. **(C)** Based on the Oncomine database, PRAP1 expression was detected in the cisplatin-treated CRC clinic cell line at 0, 6, 12, and 24 h (n=9, 12, 12 and 12).** (D)** The PARP1 protein level was evaluated by western blotting in cisplatin-resistant HCT-116 cells (HCT-116/DDP) and HT-29 cells (HT-29/DDP), 5-Fluorouracil (5-FU)-resistant HCT116 cells (HCT-116/5-Fu) and the corresponding control groups, quantitative analysis is presented on the right. *P<0.05; **P<0.01.

Based on the GEPIA 2 database, elevated PRAP1 mRNA levels were observed in patients with CRC (n=349) when compared with those in the control group (n=275) (P<0.05)** (Fig. 1A)**. Similarly, the mRNA expression of PRAP1 was also increased in different types of human CRC cell lines, including HCT-116 (P=0.0011), HT-29 (P<0.0001), LOVO (P=0.0001), and SW480 (P=0.0010) cells, when compared with that in the untransformed (normal) colon cells CRL-1790, among which the most prominent was HCT-116 cells **(Fig. 1B)**. Increased PRAP1 expression was also observed in the tumor tissues of patients with CRC when compared with that in paired adjacent normal tissues (**Fig. S1A**). However, PRAP1 expression status was not associated with the overall survival of patients with COAD (**Fig. S1B**). These results indicate that PRAP1 is highly expressed in CRC specimens, but the specific clinical research value remains unclear. Subsequently, the association between PRAP1 expression and chemotherapy resistance was assessed in patients with CRC. As shown in figure 1C, PRAP1 mRNA levels gradually declined with increasing cisplatin treatment time, according to the analysis results of Boyer CellLine database (Oncomine) **(Fig. 1C)**. In contrast, PRAP1 was more advanced in cisplatin-resistant human CRC cells (HCT-116/DDP and HT-29/DDP) and 5-Fluorouracil (5-Fu)-resistant human CRC cells (HCT-116/5-Fu) than in normal HCT-116 cells (P<0.0001; P<0.0001) and HT29 cells (P=0.0011) **(Fig. 1D)**. In addition, the Jorissen Colorectal 3 analysis showed that patients with recurrence (n=92) who had received chemotherapy displayed a higher level of PRAP1 than patients without recurrence (n=27) **(Fig. S1C)**. Combined with TCGA Colorectal analysis, the dead patients (n=19) who had received chemotherapy presented more PRAP1 expression than those who were alive (n=199) **(Fig. S1D)**. Collectively, PRAP1 might be implicated in chemotherapy resistance in CRC.

### PRAP1 regulates cisplatin-resistant CRC cells

**Figure 2:**
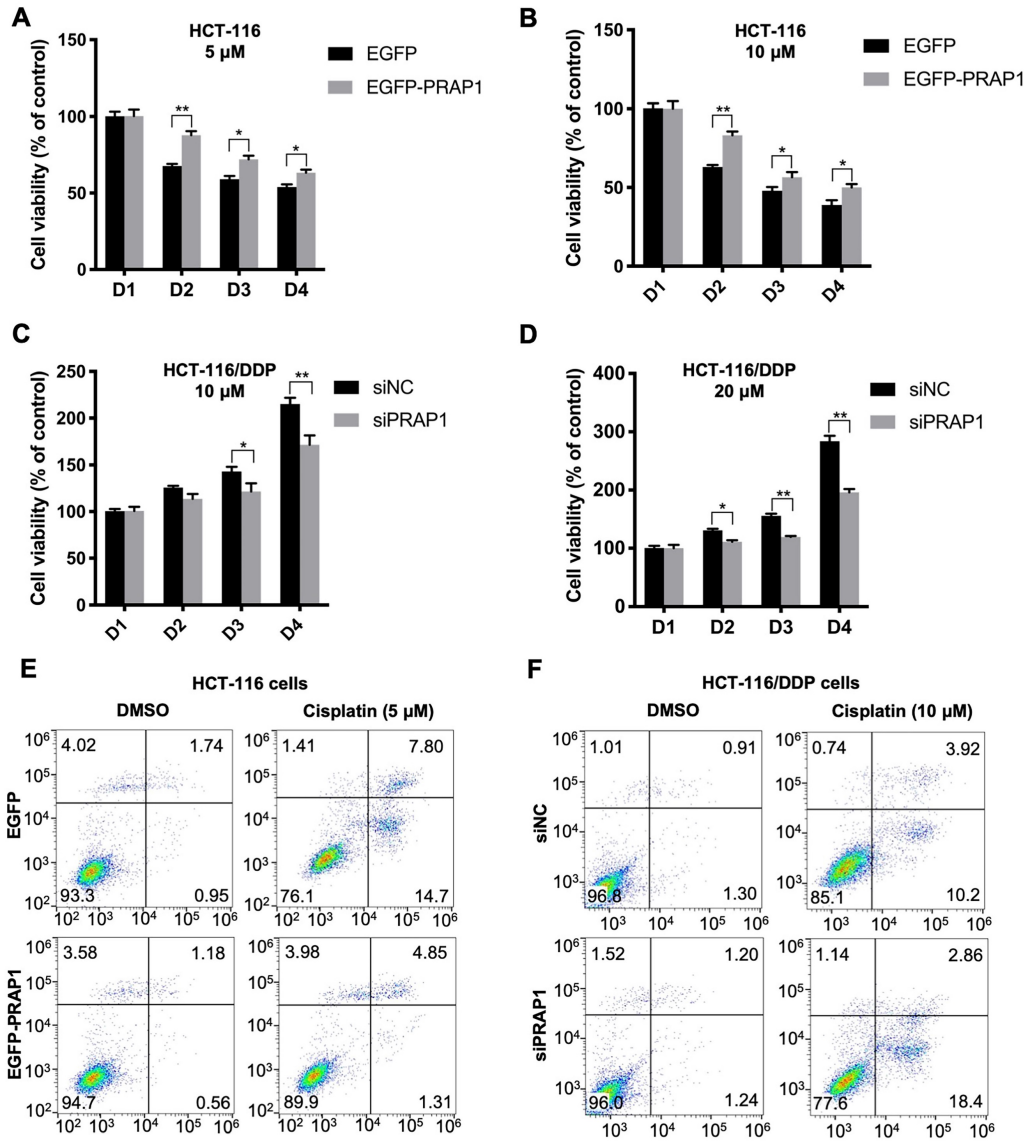
** Effects of PRAP1 on cell viability and apoptosis in HCT-116 and HCT-116/DDP cells. (A)-(B)** EGFP-PRAP1 plasmid and control vector were transfected in HCT-116 cells. Cells were induced with different concentrations of cisplatin (5 and 10 μM) for 24, 48, 72, and 96 h. Cell viability was determined by Cell Counting Kit-8 (CCK8) Assay kit. **(C-D)** siPRAP1 and negative control siRNA (siNC) were transfected in HCT-116/DDP cells. Cells were induced with 10, and 20 μM cisplatin for 24, 48, 72, and 96 h. Cell viability was monitored using CCK8 Assay kit. **(E)** EGFP-PRAP1 plasmid and control vector were transfected in HCT-116 cells, and then cells were treated with 5 μM cisplatin for 72 h. Cell apoptosis was conducted by flow cytometry using the Annexin V-FITC cell apoptosis detection kit.** (F)** siPRAP1 and siNC were transfected in HCT-116/DDP cells and cells were incubated with 10 μM cisplatin for 72 h. Cell apoptosis was detected by flow cytometry using the Annexin V-FITC cell apoptosis detection kit. *P<0.05; **P<0.01.

To demonstrate the role of PRAP1 in chemotherapy resistance in CRC, PRAP1-overexpressed HCT-116 cells and PRAP1-depleted HCT-116 cells were used. In PRAP1-overexpressed HCT-116 cells, cisplatin treatment (5 and 10 μM) significantly reduced cell viability, showing a higher inhibitory effect at a concentration of 10 μM. However, the cytotoxicity of cisplatin in HCT-116 cells was notably limited by PRAP1 overexpression (5 μM: P<0.0001, P=0.027, and P=0.0469; 10 μM: P<0.0001, P=0.015, and P=0.0378) **(Fig. 2A-2 B)**. In contrast, different concentrations of cisplatin (10 and 20 μM) had a slight effect on the viability of HCT-116/DDP cells, suggesting that HCT-116/DDP cells were resistant to cisplatin. Nevertheless, cell viability was notably reduced in cisplatin-treated HCT-116/DDP cells in the absence of PRAP1 (10 μM: P=0.0292, P<0.0001; 20 μM: P=0.0145, P=0.0014, P<0.0001)** (Fig. 2C-2D)**, suggesting that PRAP1 downregulation could sensitize cisplatin-resistant CRC cells to cisplatin. Furthermore, cisplatin-induced apoptosis was also monitored in PRAP1-overexpressed HCT-116 cells and PRAP1-depleted HCT-116 cells. As shown in figure 2E and 2F, the number of apoptotic cells increased with cisplatin (5 μM) treatment in HCT-116 cells, and the pro-apoptotic effect of cisplatin was strikingly repressed by PRAP1 upregulation **(Fig. 2E and Fig. S2A)**. Consistently, 5 μM cisplatin induced an increase in BAX and a decrease in Bcl2 levels in HCT-116 cells, which were significantly inhibited by exogenous PRAP transfection in HCT-116 cells (**Fig. S2B**). However, knockdown of PRAP1 in HCT-116/DDP cells restored cisplatin-induced apoptosis** (Fig. 2F and Fig. S2C)**. Meanwhile, 10 μM cisplatin mediated the increase of BAX, and the decrease of Bcl2 was magnified by the knockdown of PRAP1 in HCT-116/DDP cells (**Fig. S2D**). The above data show that overexpression of PRAP1 in normal HCT-116 cells results in a drug-resistant phenotype, but PRAP1 downregulation in HCT-116/DDP cells promotes the sensitivity of CRC cells to chemotherapeutic drugs.

### PRAP1 suppresses MCC assembly

**Figure 3:**
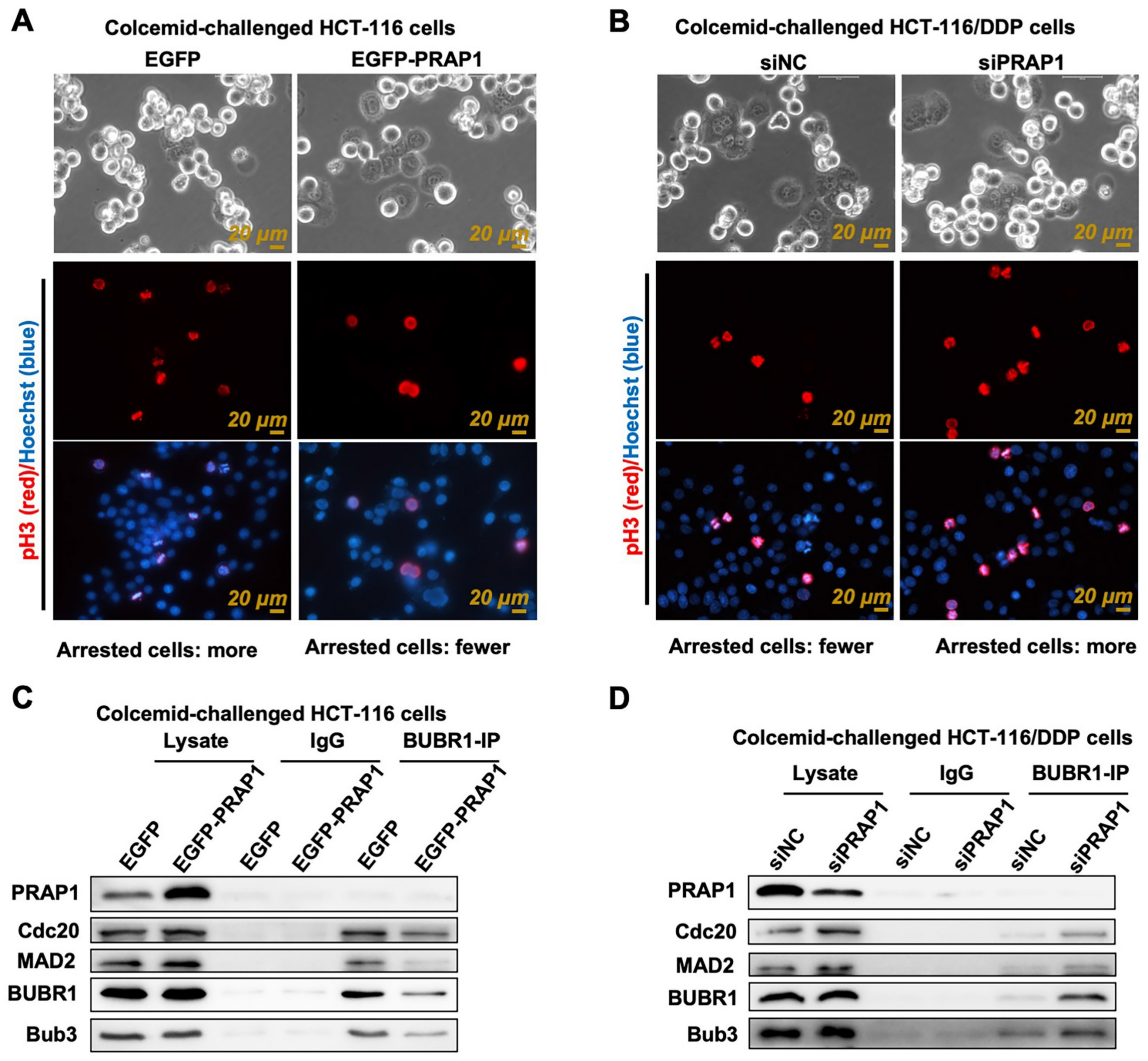
** Effect of PRAP1 on the assembly of mitotic checkpoint complex (MCC). (A)** Representative photographs of colcemid-challenged HCT-116 cells with or without EGFP-PRAP1 transfection which were examined by Livecyte Cell Analysis System. The rounded-up cell morphology was accepted to be under mitotic arrest (top). Expression and distribution of pH3-positived and apoptotic cells as monitored by IF staining. Red: pH3, Blue: Hoechst, Scale bar: 20 μm.** (B)** Representative photographs of colcemid-induced siPRAP1-treated HCT-116/DDP cells (the top picture). pH3-positive and apoptotic cells were detected by IF staining (the last two pictures). Red: pH3, Blue: Hoechst, Scale bar: 20 μm.** (C-D)** HCT-116 cells were transfected with PRAP1 recombinant overexpressed plasmid and HCT-116/DDP cells were transfected with siPRAP1. The interaction between budding uninhibited by benzimidazole-related 1 (BUBR1) with other MCC-related protein including cell-division cycle protein 20 (Cdc20), mitotic arrest deficient 2 (MAD2) and budding uninhibited by benzimidazoles 3 (Bub3) as determined by Co-IP in these cell groups.

The mitosis index is usually used to evaluate cell vitality and apoptosis, which refers to the percentage of cells in the M phase of mitosis to the total number of cells. Apoptotic cells tend to have an atypical mitosis index 28. Current methods for detecting mitotic index are based on fluorescent labeling (pH3-positive cells, a marker of nuclear division) and ultra-high-definition imaging (cells in mitosis) 29. Colcemid-challenged HCT-116 cells were distinguished using the Livecyte Cell Analysis System, and the results showed that mitotic cells had relatively round sphericity and a relatively high value of optical thickness, while many pH3-posive cells also existed in colcemid-treated HCT-116 cells, indicating that cell division was prevented **(Fig. 3A)**. However, the number of M-phase mitotic arrested cells and pH3-posive cells was reduced when PRAP1 was overexpressed in HCT-116 cells** (Fig. 3A)**. Additionally, there were few M-phase mitotic arrest cells and pH3-posive cells in colcemid-exposed HCT116/DDP cells, while the effect of colcemid rebounded after transfection of siPRAP1 in HCT116/DDP cells, showing double mitotic arrest phase cells and increased pH3-positive cells **(Fig. 3B)**. The data indicated that the colcemid-induced increase in mitosis index was abolished by PRAP1 overexpression in HCT116 cells but was elevated by PRAP1 downregulation in HCT116/DDP cells. PRAP1 may be involved in chemotherapy resistance in CRC by affecting mitosis arrest. During mitosis, improperly attached kinetochores are detected by SAC, thereby accelerating the production of MCC, which has been shown to result in drug-resistance in antitumor drugs 30, 31. Thus, the core proteins of MCC, including BUBR1, Cdc20, MAD2, and Bub3, were determined in colcemid-challenged HCT-116 cells and HCT116/DDP cells. Co-IP experiments using BUBR1 antibody showed that the interactions of BUBR1 with other MCC-related core proteins, such as Cdc20, MAD2, and Bub3, were visibly weakened in exogenous PRAP1 transfected HCT-116 cells, which indicated that the formation of MCC was blocked by PRAP1 overexpression **(Fig. 3C)**. Correspondingly, the interaction between BUBR1 and other MCC proteins was enhanced in the PRAP1-depleted HCT116/DDP cells **(Fig. 3D)**. However, no interaction was observed between MCC and PRAP1 in HCT-116 and HCT116/DDP cells. Therefore, PRAP1 negatively regulated MCC formation. It is possible that PRAP1-mediated mitotic arrest occurs by affecting MCC formation, thereby aggravating the emergence of chemotherapy resistance in CRC.

### PRAP1 induces cisplatin resistance by inhibiting MCC assembly

**Figure 4:**
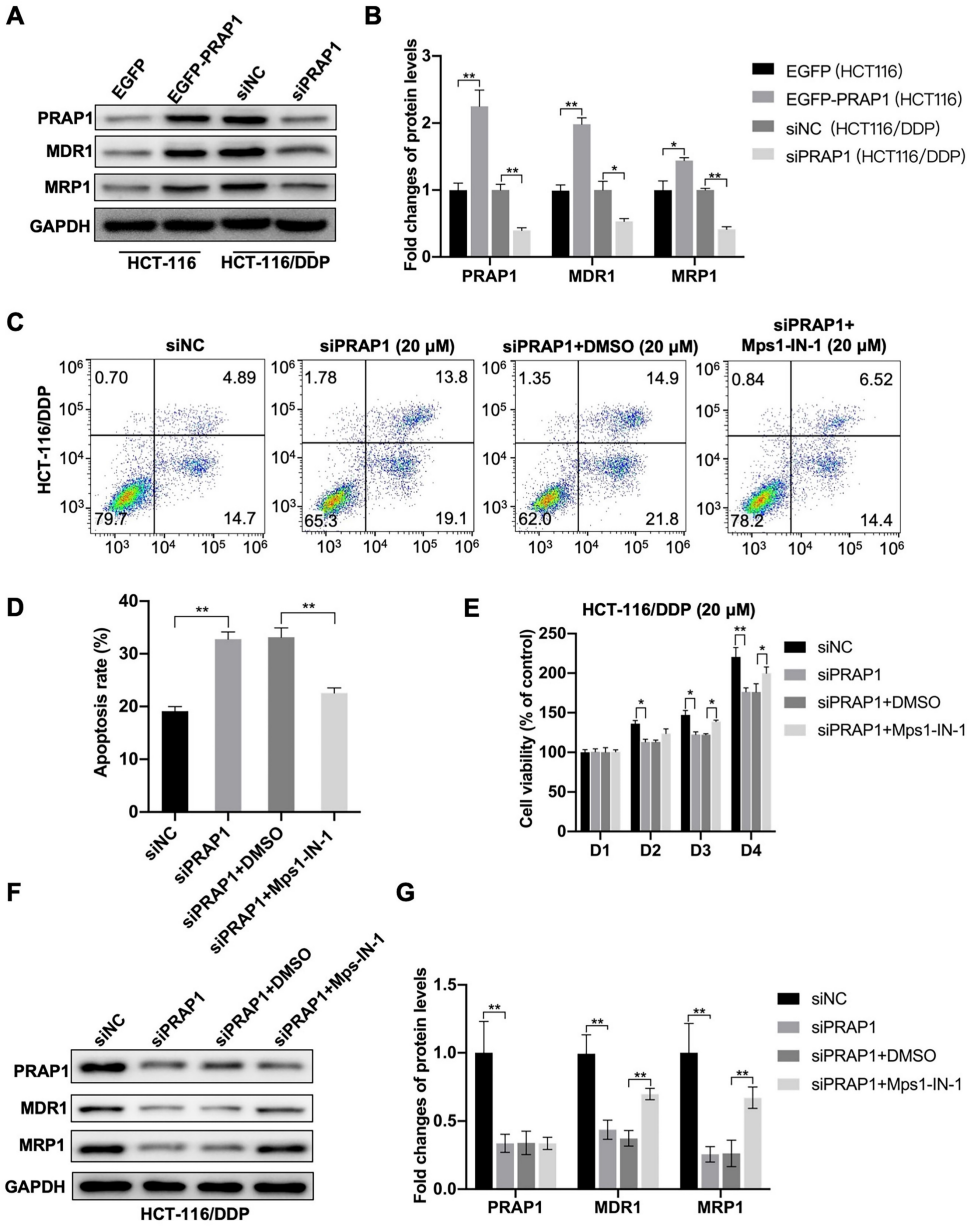
** Role of MCC in PRAP1-mediated cisplatin resistance. (A)** In PRAP1 overexpressed HCT-116 cells and siPRAP1 plasmid-transfected HCT-116/DDP cells, western blotting assay was used to detect expressions of multidrug resistant protein of p-glycoprotein 1 (MDR1) and multidrug resistance-associated protein 1 (MRP1). **(B)** Quantitative analysis of panel A as shown on the right.** (C-D)** PRAP1-depleted HCT-116/DDP cells were treated with 20 μM cisplatin and Mps1-IN-1. Cell apoptosis was evaluated by Annexin V-FITC cell apoptosis detection kit using flow cytometry (C) and quantitative analysis as presented in panel D (D). **(E)** Cell viability was measured by CCK8 Assay kit.** (F)** siPRAP1-transfected HCT-116/DDP cells were treated with 20 μM cisplatin and Mps1-IN-1, multidrug resistant protein as estimated by western blotting. **(G)** Quantitative analysis is shown on the right. *P<0.05; **P<0.01.

In PRAP1-overexpressed HCT-116 cells, increased multidrug resistance-associated protein, including MDR1 (P<0.0001) and MRP1 (P=0.0366), were observed, and decreased MDR1 (P=0.0236) and MRP1 (P=0.0038) protein levels were observed in PRAP1-depleted HCT-116/DDP cells when compared with the corresponding control cells** (Fig. 4A-4 B)**, which further demonstrate the drug resistance induced by PRAP1 overexpression. Next, the MCC assembly inhibitor Mps1-IN-1 was used to explore the role of MCC assembly in PRAP1-mediated cisplatin resistance. As shown in figure 4C, knockdown of PRAP1 in HCT-116/DDP cells promoted cisplatin-induced apoptosis, while the pro-apoptotic effect mediated by PRAP1 downregulation was abrogated by treatment with Mps1-IN-1 (P=0.0011; P=0.0066)** (Fig. 4C-4D)**. In addition, down-regulation of PRAP1 in HCT-116/DDP cells increased the inhibitory effect of cisplatin on cell viability (D2, P=0.0233; D3, P=0.0132; D4, P<0.0001). Treatment with Mps1-IN-1 reversed the cisplatin-mediated cell viability decline in PRAP1-depleted HCT-116/DDP cells (D3, P=0.01634; D4, P=0.0183) **(Fig. 4E)**. Western blotting showed that the decreased resistant proteins MDR1 (P<0.0001; P=0.0012) and MRP1 (P<0.0001; P=0.0013) mediated by PRAP1 downregulation were restored by Mps1-IN-1 challenge in HCT-116/DDP cells** (Fig. 4F-4G)**. Therefore, PARP1 depletion-mediated drug susceptibility was abolished by inhibition of MCC formation in cisplatin-resistant CRC cells.

### PRAP1 represses MCC assembly by upregulating MAD1 expression

**Figure 5:**
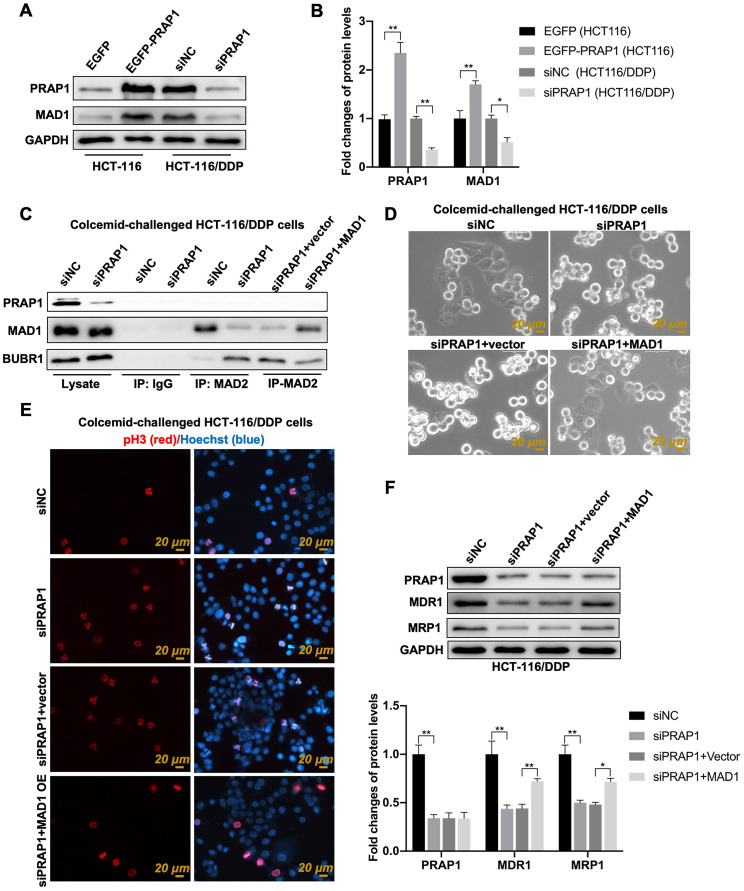
** Role of mitotic arrest deficient 1 (MAD1) in PRAP1-mediated MCC assembly inhibition and cisplatin-resistant CRC cells. (A)** HCT-116 and HCT-116/DDP cells were transfected with EGFP-PRAP1 and siPRAP1 plasmid, respectively, MAD1 expression was measured by western blotting assay. **(B)** Quantitative analysis of panel A is shown on the right. **(C)** Colcemid-challenged HCT-116/DDP cells were transfected with siPRAP1 or MAD1 overexpression plasmid, the interaction of MAD2 with MAD1, and with BUBR1 as explored by Co-IP. **(D)** Representative photographs of colcemid-challenged HCT-116/DDP cells with siPRAP1 or MAD1 overexpressing plasmid transfection which were examined using Livecyte Cell Analysis System. The rounded-up cell morphology was accepted to be under mitotic arrest (the top picture). **(E)** Expression and distribution of pH3-positive and apoptotic cells as monitored by IF staining. Red: pH3, Blue: Hoechst, Scale bar: 20 μm. **(F)** Multidrug resistant protein as estimated via western blotting and quantitative analysis as displayed below. *P<0.05; **P<0.01.

MAD1 plays an important role in MCC 32. Based on the online Cancer Biobank database, the mRNA expression of MAD1 (the gene homologous to humans was *MAD1L1*) in CRC tissues (n=275) was higher than that in the control group (n=349) **(Fig. S3A)**. Increased MAD1 expression was also found in the tumor tissues of patients with CRC when compared with that in paired adjacent normal tissues (**Fig. S3B**). Patients with COAD with high MAD1 expression status presented poor overall survival when compared with patients with low MAD1 expression status (**Fig. S3C**). Additionally, the expression of *MAD1* in CRC cell lines treated with cisplatin showed a gradient downward tendency in a time-dependent manner **(Fig. S3D)**. In 471 COAD clinical samples, *PRAP1* showed a significant positive correlation with *MAD1* **(Fig. S3E)**. Importantly, the mitotic cells had relatively round sphericity and a relatively high value of optical thickness in colcemid-treated HCT-116 cells, but the number of M-phase mitotic arrested cells was apparently reduced when MAD1 was overexpressed in HCT-116 cells** (Fig. S3F)**. The data indicated that the colcemid-induced increase in mitosis index was abolished by MAD1 overexpression in HCT116 cells, which suggested the negative regulatory effect of MAD1 on MCC assembly. Combined with the function of MAD1 during mitosis, the relationship between PRAP1 and MAD1 may be implicated in PRAP1-mediated inhibition of MCC assembly. Similarly, the transfection of exogenous PRAP1 elevated the expression of MAD1 in HCT-116 cells (P=0.0024), and PRAP1 depletion notably reduced MAD1 levels in HCT-116/DDP cells (P=0.0378) when compared with the corresponding control cells** (Fig. 5A-5B)**. Several studies have shown that MAD2 combines with BUBR1 to facilitate the orderly combination of MCC, which is essential for SAC in tumor cell apoptosis 33. In this study, the knockdown of PRAP1 in HCT-116/DDP cells significantly increased the interaction between MAD2 and BUBR1 and weakened the combination of MAD1 and MAD2; however, once MAD1 was overexpressed, the interaction between MAD2 and MAD1 strengthened and the connection between MAD2 and BUBR1 was strictly weakened in PRAP1-depleted HCT-116/DDP cells, when compared with vector plasmid-transfected HCT-116/DDP cells in the absence of PRAP1 **(Fig. 5C)**. These data suggest that PRAP1-mediated elevation of MAD1 may compete with BUBR1 to bind to MAD2, subsequently destroying the formation of MCC, resulting in escape from the supervision of MCC in HCT-116/DDP cells.

To further test this hypothesis, a colcemid-induced M-phase mitotic arrest assay was performed. PRAP1 inhibition increased the number of M-phase mitotic arrest cells and pH3-positive cells in HCT-116/DDP cells, which was abrogated by the overexpression of MAD1 in HCT-116/DDP cells **(Fig. 5D-5E)**, suggesting that the lack of PRAP1-mediated mitotic arrest was restricted by exogenous MAD1 transfection. Additionally, the expression of multidrug-resistant proteins that declined with PRAP1 inhibition in HCT-116/DDP cells was also reversed by additional MAD1 transfection (MDR1, P<0.0001, P=0.00235; MRP1, P<0.0001, P=0.0171) **(Fig. 5F)**. These implied that PRAP1-inhibited the formation of MCC via MAD1.

### PRAP1 upregulation increases the cisplatin resistance of CRC *in vivo*

**Figure 6:**
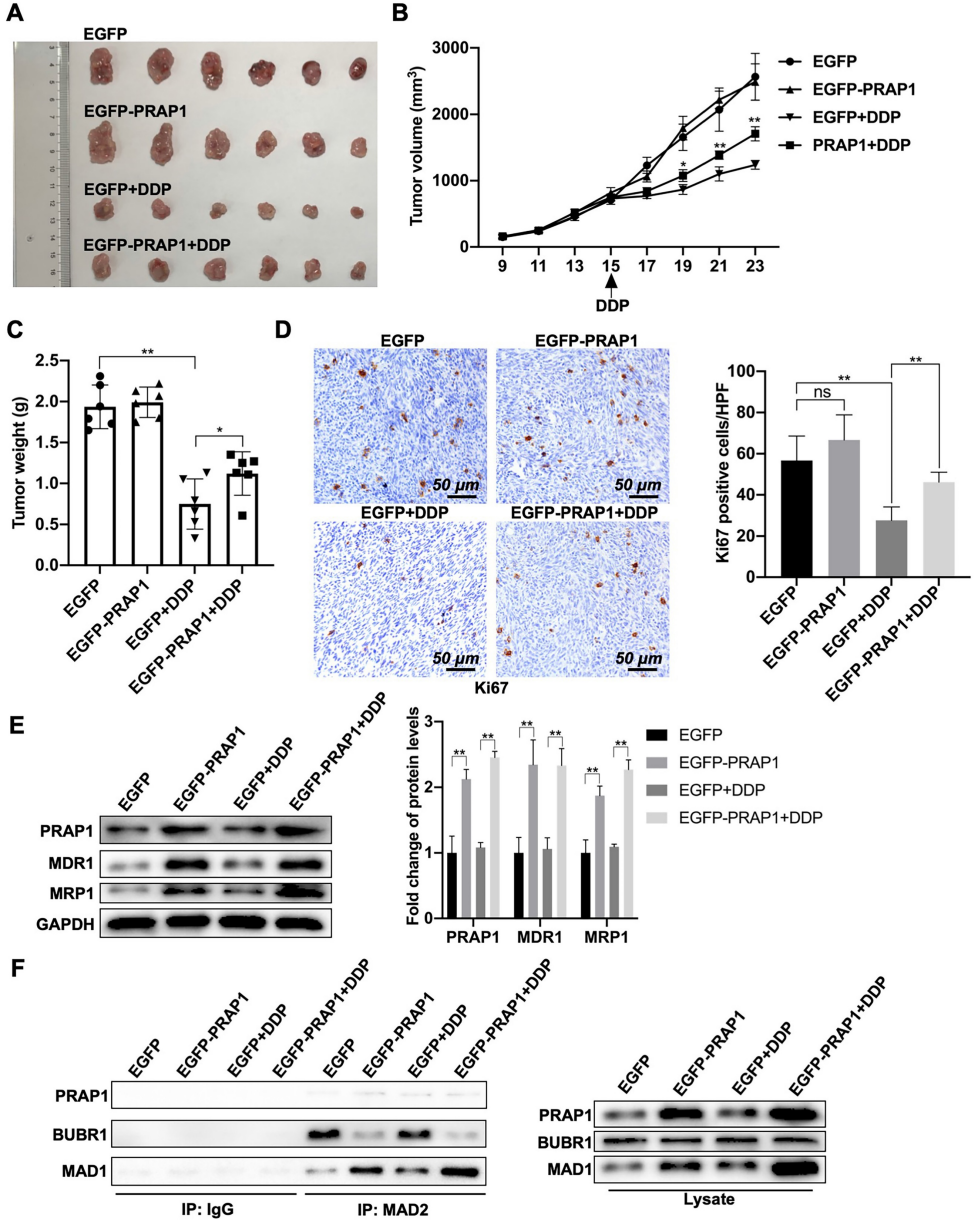
** Effects of PRAP1 on cisplatin-resistance of colorectal carcinoma *in vivo*. (A)** All mice were sacrificed by cervical dislocation and the tumors of mice from the four groups were harvested and images captured (n=6 per group).** (B)** The tumor growth curve of xenograft mice after initial cell injection and drug-treatment. Cisplatin was taken orally on day 15 after initial cell injection. **(C)** The differences in tumor weight among the four groups. **(D)** IHC staining of tumors with Ki67 antibody in the four groups. Relative quantitative analysis of Ki67-positive cell number is shown in the left panel. Scale bar, 50 μm. **(E)** Drug resistance related protein levels including MDR1 and MAD1, and protein level of PRAP1 in tumors as determined by western blotting. Relative quantitative analysis of protein levels is shown on the left panel. **(F)** The interaction of MAD2 with MAD1, and with BUBR1 in tumors as explored by Co-IP. ns, no significant. * P<0.05, ** P<0.01.

Next, the antitumor effects of cisplatin in tumor-bearing nude mice were examined. A mouse tumor model was generated by the subcutaneous injection of control HCT-116 cells and PRAP1-overexpressed HCT-116 cells. Once the tumor volume reached approximately 1 cm^3^, nude mice bearing tumors were treated with saline or cisplatin. When compared with the control group (EGFP), there was no significant change in tumor growth in the PRAP1 overexpression group. Compared with saline-treated mice, tumors from both the control and PRAP1-overexpression groups were significantly suppressed after cisplatin treatment. However, tumors in the PRAP1-overexpression group showed weaker sensitivity to doxorubicin administration than tumors in the control HCT-116 group. Briefly, the tumor volume of the PRAP1-overexpression group treated with cisplatin was larger than that of the control group **(Fig. 6A)**. Additionally, the tumor growth curve visually showed that mice injected with PRAP1-overexpressing HCT-116 cells developed resistance to cisplatin in comparison with the control group mice (P=0.0192; P=0.0006; P<0.0001) **(Fig. 6B)**. The tumor weight loss caused by drug treatment was lower due to PRAP1 overexpression (P<0.0001; P=0.0485) when compared with the cisplatin-treated control mice** (Fig. 6C)**. However, no growth curve or body weight differences were detected between the control group and PRAP1-overexpressing mice without drug treatment** (Fig. 6B-6C).**

In tumor tissues, Ki67-positive cells were moderately elevated (not significant) in the PRAP1-overexpression group when compared with those in control mice without drug treatment (P=0.0004; P=0.0002) **(Fig. 6D)**. After cisplatin treatment, the number of Ki67-positive cells decreased sharply in control mice, whereas it was notably restored in the PRAP1-overexpression group after cisplatin challenge **(Fig. 6D)**. In addition, higher drug resistance-related proteins, including MDR1 (P<0.0001; P<0.0001) and MRP1 (P<0.0001; P<0.0001), were observed in tumors of the PRAP1-overexpression group, which were further elevated after cisplatin administration **(Fig. 6E)**. Consistently, PRAP1 overexpression significantly elevated the interaction between MAD1 and MAD2 and weakened the interaction between BUBR1 and MAD2. Interestingly, cisplatin treatment moderately (but not significantly) increased the binding capability of MAD2 to MAD1, but decreased the interaction between BUBR1 and MAD2 (no significant difference). When compared with cisplatin-challenged control mice, more interactions between MAD1 and MAD2 and less interactions between BUBR1 and MAD2 were found in cisplatin-challenged PRAP1 overexpression mice **(Fig. 6F)**. These data further confirmed that PRAP1 upregulation increased the interaction between MAD1 and MAD2 and competitively inhibited the binding of BUBR1 to MAD2 *in vivo*, resulting in MCC formation disorder and the formation of cisplatin-resistant tumors. Therefore, PRAP1 is involved in MCC destruction-mediated cisplatin resistance in colorectal carcinoma.

## Discussion

Although there has been encouraging progress in CRC therapy, the survival rate is frequently compromised by chemoresistance 34. Checkpoint inhibitors SAC and MCC participate in escape mechanisms for chemotherapeutic drug resistance 35. This study is aimed to reveal the possible mechanisms underlying cisplatin resistance in CRC. In cisplatin-resistant CRC cells, PRAP1 manages MAD1, which can bind to MAD2, an important molecule in MCC, blocking the integrity of MCC and causing SAC abnormalities. Our findings provide a novel strategy for the clinical treatment of cisplatin-resistant CRC.

PRAP1 is expressed in the liver, gastrointestinal tract, and kidneys and can control tumor cell growth in hepatocellular carcinoma and bladder cancer 25, 36. In this study, high levels of PRAP1 were observed in CRC patients and a variety of CRC cell lines. Possibly, abnormal PRAP1 expression may be involved in the development of CRC. Cisplatin is adopted for the treatment of many malignancies such as ovarian, head and neck, colorectal and lung cancers 37, 38. In our study, PRAP1 expression in CRC cells was decreased with prolonged cisplatin-induced treatment. Notably, patients with CRC with high PRAP1 levels have a higher recurrence rate and mortality after receiving chemotherapy. Hence, we can reasonably conclude that PRAP1 is involved in cisplatin resistance in CRC. P53 can increase the level of PRAP1, thereby enhancing chemotherapeutic drug resistance because the increase in PRAP1 promotes the proliferation of tumor cells 39. After irradiation induction, Prap1^-/-^ mice showed notably increased p21 and cell apoptosis in the small intestinal epithelium 40. These results demonstrate that PRAP1 can regulate drug resistance by affecting cell viability and apoptosis. In HCT-116 cells, PRAP1 upregulation promoted cisplatin resistance* in vitro* and *in vivo*, and downregulation of PRAP1 in HCT-116/DDP cells enhanced chemosensitivity. This implies that overexpression of PRAP1 in normal CRC cells results in resistance to chemotherapeutic drugs.

The function of the SAC is to ensure that all chromosomes are correctly connected to the spindle power point so that the chromosomes can be separated correctly 41, 42. SAC regulates mitotic-arrest through MCC complex, which contains several related proteins that regulate mitosis, including Cdc20, MAD2, BUBR1, MAD3, and Bub3 43-45. Mitotic index, that cells in the mitotic arrest phase or pH3-assessed mitotic cells, are positively correlated with apoptosis 46. Cells with a higher mitotic index can't proliferate normally and enter apoptotic events 28, 47. In our study, we found that PRAP1 could eliminate colcemid-induced mitotic arrest, and the powerless interactions between BUBR1 and other MCC molecules. Thus, the PRAP1-mediated reduction in colcemid-induced mitotic arrest may be due to the failure of MCC assembly.

High expression of MDR1 and MRP1 is responsible for multidrug resistance in many cancers 48, 49. Clinical retrospective analysis showed that the levels of MDR1, MRP1, and BCR1 in chemoresistant CRC are significantly higher than those in normal colorectal patients 50. Inhibition of the spindle checkpoint protein expression of BUBR1 and MAD2 increases tumor resistance to paclitaxel and doxorubicin, causing a reduction in MDR1 and MRP1 51. Thus, correct MCC assembly plays a determinant role in drug resistance in tumor cells. Since PRAP1 overexpression mediates drug resistance in CRC cells, the underlying mechanism may have implications for MCC assembly. In the present study, PRAP1 inhibition-caused the chemosensitivity to cisplatin in HCT-116/DDP cells was blocked by limiting the formation of MCC. In addition, the PRAP1 removal-mediated decline in multidrug-resistant protein expression was also reversed by a specific inhibitor of MCC assembly. Our findings suggest that PRAP1 negatively affects MCC activity and mediates cisplatin resistance. We first shed light on the function of PRAP1 in the chemotherapy resistance of CRC, but the comprehensive resistance mechanism and the role of MCC assembly in these processes require further elucidation.

CRC cells produce doxorubicin resistance to chemotherapy under hypoxic conditions, mainly because MAD1 inhibits the ROS response produced by mitochondria 52. A case-control study of patients with CRC uncovered a mutation in the protein domain of MAD1, especially *MAD1L1* Arg558His, which causes susceptibility to CRC 53. These data indicated that MAD1 may inhibit chemotherapeutic drug resistance in CRC. Interestingly, yeast two-hybrid screening suggests that PRAP1 interacts with MAD1 to induce SAC impairment in hepatocellular carcinoma 25. Given that the impairment of SAC formation gives rise to drug resistance, this finding implies that the interaction between PRAP1 and MAD1 may affect the chemotherapy resistance of cancer cells. Our research indicates a high expression of MAD1 and a significant association between PRAP1 and MAD1 in CRC tumors. Both MAD1 and MAD2 are located in nuclear pores and can competitively bind to Cdc20, exerting opposite effects 54. It is generally believed that MAD2 combines with Cdc20 to form the MAD2-Cdc20 complex, which impedes the anaphase-promoting complex that further supports the separation of chromosomes during mitosis 55. Nevertheless, the combination of MAD1 and Cdc20 reverses this phenomenon, thus allowing cells to enter subsequent divisions 56. Downregulation of MAD2 and BUBR1 in SiHa cells creates a SAC defect, subsequently causing malignant proliferation and nocodazole resistance 57. In the present study, PRAP1 knockdown reduced the interaction between MAD2 and MAD1, but promoted the interaction between MAD2 and BUBR1 in HCT-116/DDP cells. Simultaneously, MAD1 upregulation also affected PRAP1 removal-mediated elevation in colcemid-induced mitotic arrest. It is possible that the PRAP1-induced expression of MAD1 competitively integrates MAD2 to limit MCC formation. Although MAD1 has been found to play a role in chemotherapy resistance in CRC and ovarian cancer 52, 58, its effect on multidrug resistance in cancer chemotherapy has rarely been reported. Surprisingly, we found that MAD1 upregulation abolished the PRAP1 inhibition-mediated improvement in drug resistance in PRAP1-depleted HCT-116/DDP cells. This result indicates that PRAP1 facilitates the expression of multidrug-resistant proteins by modulating MAD1 and the interaction between MAD1 and MAD2. However, whether MAD1 can competitively bind to other core molecules of MCC to disrupt MCC assembly remains unclear. In addition, large-scale clinical trials are required to confirm our findings.

## Conclusion

**Figure 7:**
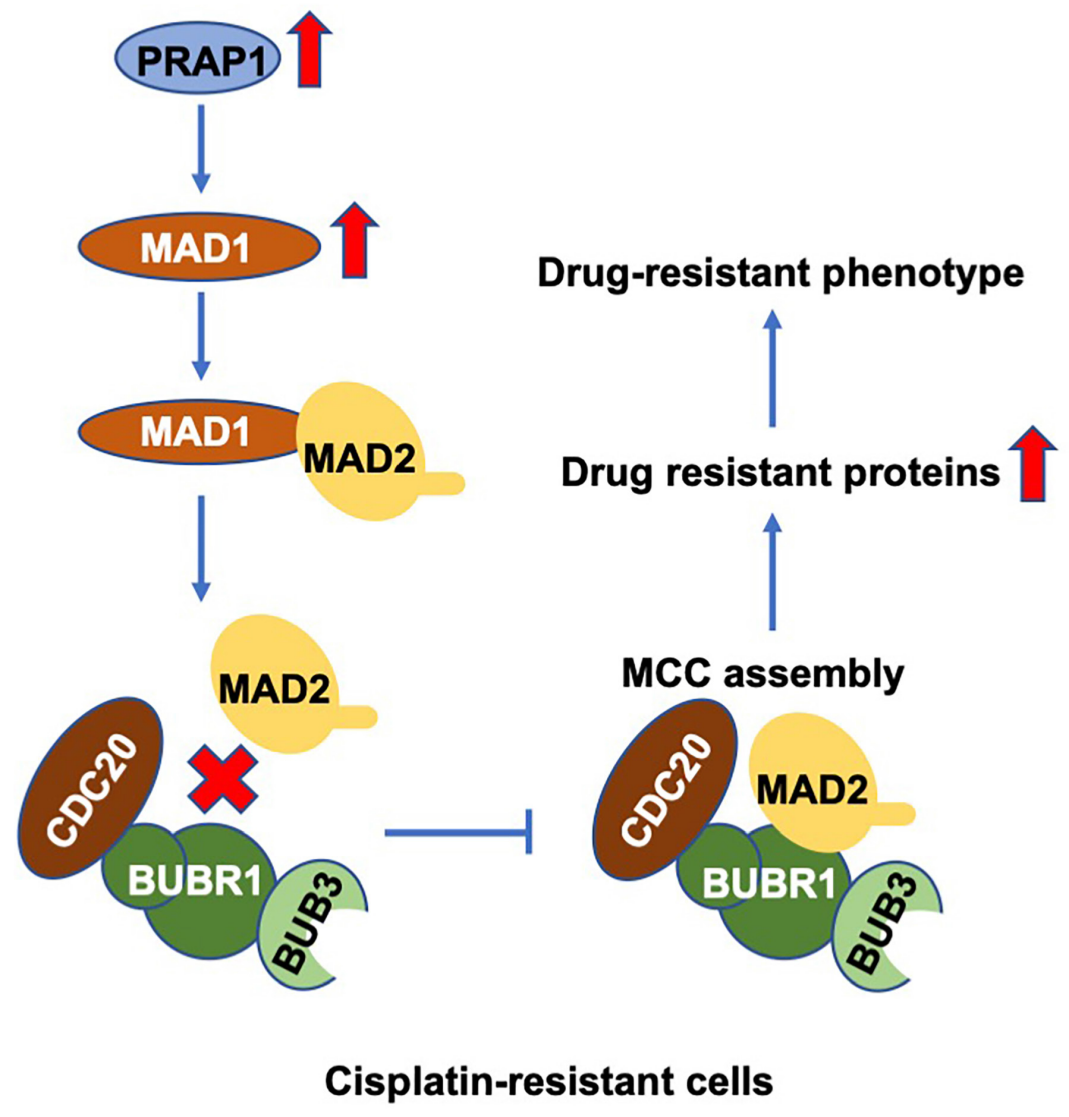
** Schematic diagram of PRAP1-mediated cisplatin-resistance.** PRAP1 overexpression caused the cisplatin resistance in CRC. Mechanically, PRAP1 could positively regulate MAD1 expression, and the binding of MAD1 to MAD2 destroyed MCC assembly by weakening the interaction between MAD2 and BUBR1, which in turn allowed tumor cells to escape from the control of MCC, resulting in the drug-resistant phenotype.

In summary, PRAP1 is responsible for cisplatin resistance, possibly by inhibiting MCC formation in CRC. Mechanistically, PRAP1 positively regulate MAD1 expression, and the binding of MAD1 to MAD2 destroyed MCC assembly by weakening the interaction between MAD2 and BUBR1, which in turn allowed tumor cells to escape from the control of SAC **(Fig. 7)**. These findings provide a novel perspective for discovering the underlying molecular mechanisms and exploring new therapeutic targets for chemotherapy-resistant CRC.

## Supplementary Material

Supplementary figures and table.

## Figures and Tables

**Table 1:** Primer sequences of RT-PCR.

Gene	Forward	Reverse
***PRAP1***	5'-ACTCTCTACAGAGACGCGGA-3'	5'-TCTGAGGGCCAGTGTTTGAC-3'
***GAPDH***	5'-ATCACCCCACTTTACCCCTC-3'	5'-TTTTGTCTCGGCTGTTTCGG-3'

## Abbreviations

PRAP1: Proline-rich acidic protein 1
MAD1: mitotic arrest deficient 1
MCC: mitotic checkpoint complex
CRC: colorectal carcinoma
MRP1: multidrug resistance-associated protein 1
MDR1: p-glycoprotein 1
MAD2: mitotic arrest deficient 2
SAC: spindle assembly checkpoint
Cdc20: cell-division cycle protein 20
BUBR1: budding uninhibited by benzimidazole-related 1
Bub3: budding uninhibited by benzimidazoles 3
COAD: colon adenocarcinoma
5-FU: 5-Fluorouracil
