# Supplementary material for: Proline-rich acidic protein 1 upregulates mitotic arrest deficient 1 to promote cisplatin-resistance of colorectal carcinoma by restraining mitotic checkpoint complex assembly
Source: J Cancer. 2023 May 21;14(9):1515–30. doi: 10.7150/jca.84048 (PMC10266255; doi:10.7150/jca.84048)
Supplement: Supplementary file 1 — Supplementary figures and table. [file jcav14p1515s1.pdf]

Supplementary materials

Supplementary Figure legends

Figure S1

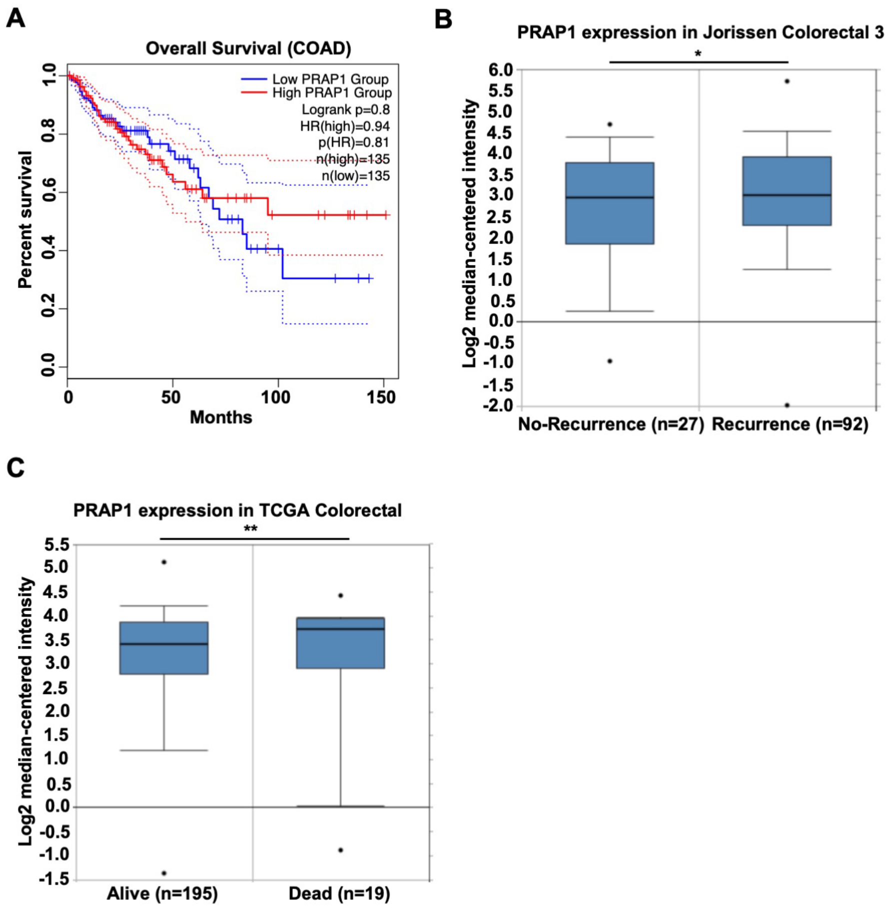

Figure S1 PRAP1 expression in patients with CRC receiving chemotherapy. (A) Human tumor tissues of patients with CRC (n=11) were collected. The expression of PRAP1 as determined by IHC staining. Scale bar, 50 μm. **(B)** Overall survival of COAD based on PRAP1 expression status as analyzed by GEPIA 2.0. **(C)** Based on Oncomine database (Jorissen Colorectal 3), the PRAP1 expression was analyzed in recurrent (n=92) and non-recurrent (n=27) patients with CRC who received chemotherapy. **(D)** Based on Oncomine database (TCGA Colorectal), the PRAP1 expression was detected in alive (n=195) and dead (n=19) patients with CRC who received chemotherapy. *P < 0.05; **P < 0.01.

Figure S2

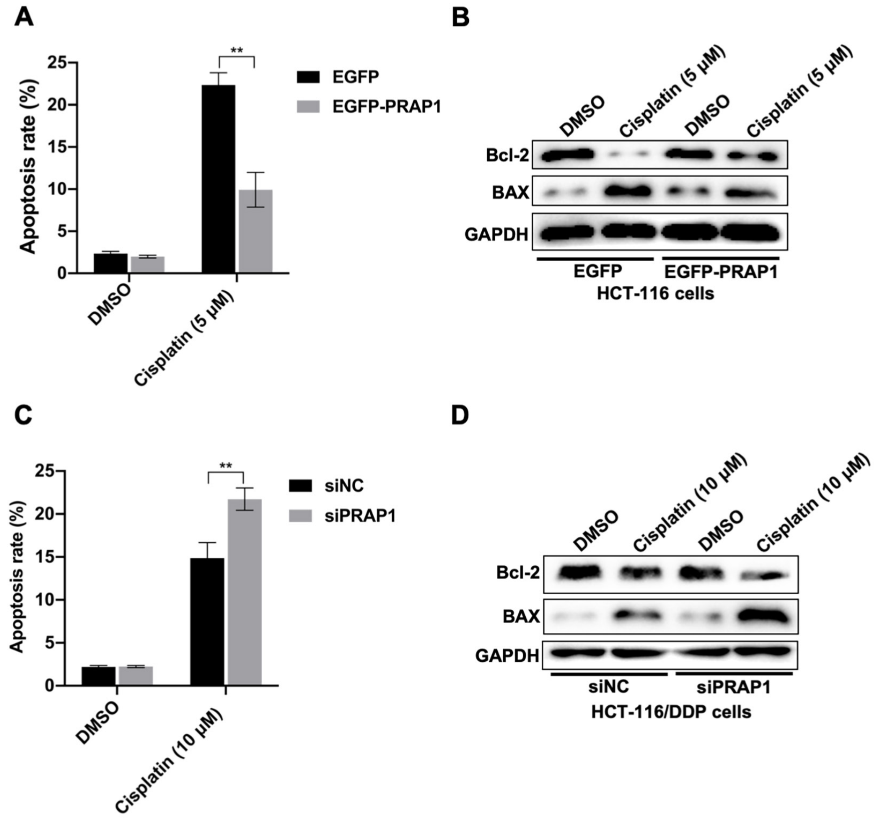

Figure S2 Effect of PRAP1 on cell apoptosis in HCT-116 cells and HCT-116/DDP cells. (A) Quantitative analysis of cell apoptosis in cisplatin-treated HCT-116 cells with or without PRAP1 transfection. **(B)** Protein levels of Bcl-2 and BAX as determined by western blotting in cisplatin-treated HCT-116 cells with or without PRAP1 transfection. **(C)** Quantitative analysis of cell apoptosis in cisplatin-treated HCT-116/DDP cells with or without PRAP1 transfection. **(D)** Protein levels of Bcl-2 and BAX as determined by western blotting in cisplatin-treated HCT-116/DDP cells with or without PRAP1 transfection. **P < 0.01.

Figure S3

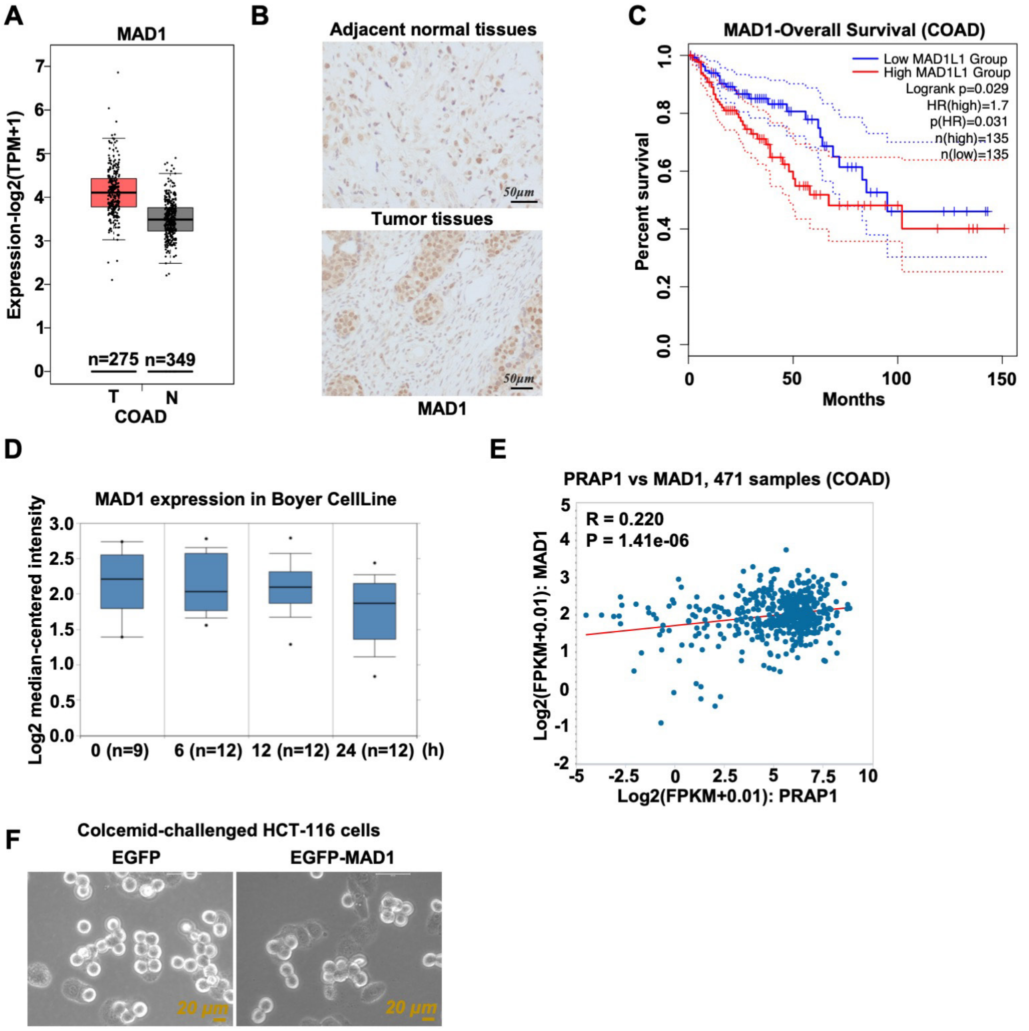

Figure S3 Expression pattern of MAD1 in CRC specimens and the role of MAD1 on mitotic arrest. (A) MAD1 expression in patients with CRC (n=275) and control group (n=349) as analyzed by GEPIA 2. **(B)** Human tumor tissues of patients with CRC (n=11) were collected. The expression of MAD1 as determined by IHC staining. Scale bar, 50 μm. **(C)** Overall survival of COAD based on PRAP1 expression status as analyzed by GEPIA 2.0. **(D)** PRAP1 expression was detected in the cisplatin-treated CRC clinic cell line at 0, 6, 12, and 24 h (n=9, 12, 12 and 12). **(E)** Expression association between PRAP1 and MAD1L1 was analyzed using Encyclopedia of RNA Interactomes (ENCORI) Starbase (n=471). **(F)** Representative photographs of colcemid-challenged HCT-116 cells with or without EGFP-MAD1 transfection which were examined by Livecyte Cell Analysis System. The rounded-up cell morphology was accepted to be under mitotic arrest (top). *$P < 0.05$; **$P < 0.01$.

Table S1 Clinic parameters of enrolled colorectal carcinoma patients.

Number	Gender	Age	Pathology	Anatomic_neoplasm
1	Male	63	Moderately differentiated colorectal carcinoma	Ascending Colon
2	Male	55	Moderately differentiated colorectal carcinoma	Transverse Colon
3	Male	47	Highly differentiated colorectal carcinoma	Ascending Colon
4	Male	72	Highly differentiated colorectal carcinoma	Hepatic Flexure
5	Female	61	Highly differentiated colorectal carcinoma	Ascending Colon
6	Male	58	Moderately differentiated colorectal carcinoma	Ascending Colon
7	Male	43	Moderately differentiated colorectal carcinoma	Transverse Colon
8	Male	70	Poorly differentiated colorectal carcinoma	Ascending Colon
9	Male	65	Highly differentiated colorectal carcinoma	Transverse Colon
10	Male	52	Poorly differentiated colorectal carcinoma	Ascending Colon
11	Male	59	Moderately differentiated colorectal carcinoma	Ascending Colon
